# Autologous platelet-rich gel and continuous vacuum sealing drainage for the treatment of patients with diabetic foot ulcer: Study Protocol

**DOI:** 10.1097/MD.0000000000017928

**Published:** 2019-11-15

**Authors:** Jie Xu, Qiao-Yun Wang, Wei Li

**Affiliations:** a Department of Endocrine and Metabolism; b Department of Ultrasound Diagnosis, Yan’an University Affiliated Hospital, Yan’an, China.

**Keywords:** autologous platelet-rich gel, continuous vacuum sealing drainage, diabetic foot ulcer, efficacy, safety

## Abstract

**Background:**

Research focusing on the efficacy of autologous platelet-rich gel (APRG) and continuous vacuum sealing drainage (CVSD) for diabetic foot ulcer (DFU) is increasing. Despite increasing knowledge on this theme, its results remain inconsistent. Thus, we will provide insight into the efficacy of APRG and CVSD for patients with DFU.

**Methods:**

We will search electronic databases of MEDILINE, EMBASE, Cochrane Library, CINAHL, AMED, Chinese Biomedical Literature Database, and China National Knowledge Infrastructure from inception to October 1, 2019. No language limitation is utilized to these databases. Two authors will independently perform study selection, data extraction, and risk of bias assessment. Disagreements between 2 authors will be solved through discussion with a third author.

**Results:**

The efficacy and safety of APRG and CVSD for patients with DFU will be assessed by the time to complete healing, proportion of ulcers healed within trial period, change of size of ulcer, health-related quality of life, patient length of hospital stay, and adverse events.

**Conclusion:**

The results of this study will provide helpful evidence of APRG and CVSD for patients with DFU.

**Systematic review registration:**

PROSPERO CRD42019153289.

## 1 Introduction

Diabetic foot ulcer (DFU) is one of the most common and severe complications in patients with diabetes mellitus (DM).^[1–4]^ Such condition has increased dramatically over the past decades around the world.^[5–7]^ It affects 15% of DM patients during their lifetime.^[8,9]^ Previous studies have found that DFU is associated with higher incidence of infection, gangrene, amputation, and morbidity.^[10–14]^ Thus, early effective managements of DFU can greatly decrease the severity of complications including poor quality of life, amputations, and even death.^[15–18]^

In recent years, there is an increasing interest in the encouraging role of autologous platelet-rich gel (APRG) and continuous vacuum sealing drainage (CVSD) for patients with DFU.^[18–26]^ However, no study systematically assesses its efficacy and its results are still unclear. Therefore, this study will investigate the efficacy and safety of APRG and CVSD for the treatment of patients with DFU at the evidence-based medicine level.

## 2 Methods and analysis

### 2.1 Dissemination and ethics

We will publish this study at a peer-reviewed scientific journal. No formal ethical approval or informed consent is inquired, because we will not utilize individual patient data.

### 2.2 Eligibility criteria

#### 2.2.1 Types of studies

We will consider all randomized controlled trials (RCTs) that investigated the efficacy of APRG and CVSD for patients with DFU. We will remove any studies, except RCTs.

#### 2.2.2 Types of participants

We will include all patients who diagnosed with DFU with no limitations of their race, sex, and age.

#### 2.2.3 Types of interventions

In the experimental group, we will select all trials assessing the efficacy and safety of APRG and CVSD for DFU.

In the control group, we will choose all studies using all other therapies, except APRG or CVSD, or APRG combined CVSD.

#### 2.2.4 Types of outcome measurements

Primary outcomes include time to complete healing, and proportion of ulcers healed within trial period.

Secondary outcomes consist of change of size of ulcer; health-related quality of life; patient length of hospital stay, and adverse events.

### 2.3 Search methods for the identification of studies

#### 2.3.1 Electronic database searches

Electronic database searches will be conducted for potential studies from MEDILINE, EMBASE, Cochrane Library, CINAHL, AMED, Chinese Biomedical Literature Database, and China National Knowledge Infrastructure from their inceptions to October 1, 2019. We will not impose language limitation to these databases. The MEDLINE search strategy is exerted in Table 1. Similar search strategies will be adapted for other electronic databases.

**Table 1:**
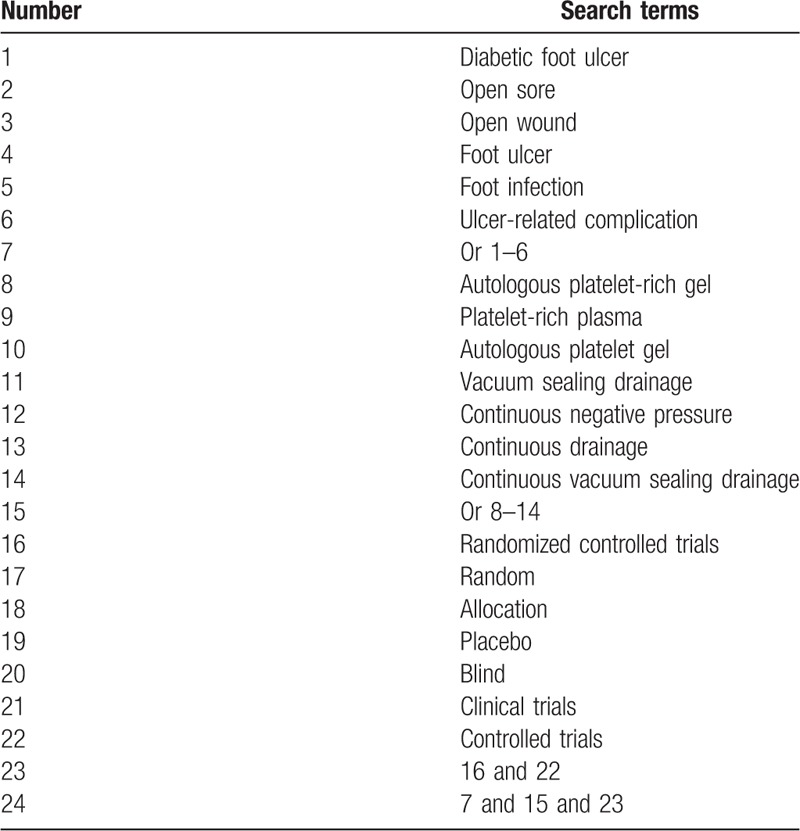
Search strategy for MEDLINE.

#### 2.3.2 Other resources searches

Aside from above electronic databases, we will also search conference proceedings, clinical registry, and reference lists of associated reviews.

### 2.4 Data collection and analysis

#### 2.4.1 Screening of the studies

Two authors will independently scan all the retrieved titles and abstracts based on the predefined and determined eligibility criteria. All irrelevant literatures will be excluded. Full text of all remaining studies will be screened to further confirm the final selection of eligible studies. All reasons for exclusion of each study will be noted. In case of any divergences, a third author will be referred to make a final consistent decision. The process of study selection will be exerted and presented in the flowchart.

#### 2.4.2 Data extraction

Two authors will perform the data extraction according to the predefined data extraction form. Divergences between 2 authors will be resolved by a third author to reach consensus if necessary. The extracted data consists of first author, title, time of publication, patient characteristics (sample size, inclusion and exclusion criteria, age, gender, et al), study methods (random methods, blind, allocation, et al), interventions (type of intervention, dosage, duration, frequency, et al), outcome measurements (primary, secondary and safety outcomes), and conflict of interest.

#### 2.4.3 Risk of bias assessment

The risk of bias assessment of included studies will be assessed by using Cochrane Handbook for Systematic Reviews of Interventions tool. It covers 7 aspects, and each one is also divided as high, unclear and low risk of bias. Two authors will perform these judgments of risk of bias, and any different opinions will be solved by discussion with the help of a third author as an arbitrator if necessary.

#### 2.4.4 Dealing with missing data

When there is unclear or insufficient information, we will contact primary authors to require it. If such information cannot be inquired, we will analyze available data.

#### 2.4.5 Data synthesis

We will use RevMan 5.3 software to perform statistical analysis. For enumeration data, we will use risk ratio and 95% confidence intervals (CIs) to express it. For continuous data, we will exert mean difference or standardized mean difference with 95% CIs to present it. We will identify heterogeneity of eligible studies by using *I*^2^ statistic. *I*^2^ ≤ 50% exerts acceptable heterogeneity, and a fixed-effects model will be used. Meanwhile, we will conduct meta-analysis. *I*^*2*^ > 50% indicates obvious significant heterogeneity, and a random-effect model will be applied. At the same time, we will perform subgroup analysis to explore the possible reasons for such obvious substantial heterogeneity. Whenever significant heterogeneity still exerts, we will carry out narrative synthesis to present the findings, structured around intervention types, target participant characteristics, and types of interventions and outcomes.

#### 2.4.6 Reporting bias

We will perform funnel plot and Egger regression test if more than 10 eligible RCTs will be included.^[27]^

#### 2.4.7 Subgroup analysis

We will conduct subgroup analysis according to the different study or patient characteristics, interventions, controls and outcome measurements.

#### 2.4.8 Sensitivity analysis

We will operate sensitivity analysis to check robustness of results by excluding high risk of bias studies.

## 3 Discussion

To the best of our knowledge, this study firstly concentrates on the efficacy and safety of APRG and CVSD for DFU. This study attempts to perform a comprehensive analysis of the existing evidence to fill this gap in the research domain. The findings of this study will provide a detailed summary of the present evidence of APRG and CVSD for treatment of patients with DFU. This study will also provide guidance for both clinical practice and further research.

## Author contributions

**Conceptualization:** Jie Xu, Qiao-yun Wang, Wei Li.

**Data curation:** Jie Xu, Qiao-yun Wang, Wei Li.

**Formal analysis:** Jie Xu.

**Investigation:** Qiao-yun Wang, Wei Li.

**Methodology:** Jie Xu.

**Project administration:** Wei Li.

**Resources:** Jie Xu, Qiao-yun Wang.

**Software:** Jie Xu, Qiao-yun Wang.

**Supervision:** Wei Li.

**Validation:** Jie Xu, Qiao-yun Wang, Wei Li.

**Visualization:** Jie Xu, Qiao-yun Wang, Wei Li.

**Writing – original draft:** Jie Xu, Qiao-yun Wang, Wei Li.

**Writing – review & editing:** Jie Xu, Qiao-yun Wang, Wei Li.
